# Foundation Pattern, Productivity and Colony Success of the Paper Wasp, *Polistes versicolor*

**DOI:** 10.1673/031.010.12501

**Published:** 2010-08-05

**Authors:** Simone Alves de Oliveira, Mariana Monteiro de Castro, Fábio Prezoto

**Affiliations:** Programa de Pós-Graduação em Ciências Biológicas - Comportamento e Biologia Animal, Laboratório de Ecologia Comportamental, Universidade Federal de juiz de Fora, Campus Universitário, Martelos, Juiz de Fora, Minas Gerais State, Brazil. Cep 36036-900.

**Keywords:** anthropic environments, foundresses, independent founding, nesting behavior, social wasps

## Abstract

*Polistes versicolor* (Olivier) (Hymenoptera: Vespidae) colonies are easily found in anthropic environments; however there is little information available on biological, ecological and behavioral interactions of this species under these environmental conditions. The objective of this work was to characterize the foundation pattern, the productivity, and the success of colonies of *P. versicolor* in anthropic environments. From August 2003 to December 2004, several colonies were studied in the municipal district of Juiz de Fora, Southeastern Brazil. It was possible to determine that before the beginning of nest construction the foundress accomplishes recognition flights in the selected area, and later begins the construction of the peduncle and the first cell. As soon as new cells are built, the hexagonal outlines appear and the peduncle is reinforced. Foundation of nests on gypsum plaster was significantly larger (p < 0.0001; χ^2^ test) in relation to the other types of substrate, revealing the synantropism of the species. On average, the *P. versicolor* nest presents 244.2 ± 89.5 (100–493) cells and a medium production of 171.67 ± 109.94 (37–660) adults. Cells that produced six individuals were verified. Usually, new colonies were founded by an association of females, responsible for the success of 51.5%. Although these results enlarge knowledge on the foundation pattern of *P. versicolor* in anthropic environments, other aspects of the foundation process require further investigation.

## Introduction

The neotropical social wasp, *Polistes versicolor* (Olivier) (Hymenoptera: Vespidae), possesses nests consisting of a single comb fixed to the substratum by a peduncle (Richards 1978). The simple arrangement of suspended cells seems to protect the colony from ant attacks, which constitute the largest predatory pressure for social wasps (Jeanne 1975, 1980; Post and Jeanne 1985).

*P. versicolor* colonies can be found in different types of substrata such as leaves, branches, roots, stones, and also in abandoned nests of other social wasp species. The nests are built with vegetable material, chewed and mixed with secretion of salivary glands, and the peduncle is resinous (West-Eberhard 1969; Spradbery 1973; Reeve 1991). In anthropic areas, the presence of nests using several structures as nesting substrata have been observed (Fowler 1983; Giannotti 1992; Lima et al. 2000; Prezoto 2001; Prezoto et al. 2007). However, the behavior and the biology of this species in the anthropic environment arelittle understood.

During nest foundation the solitary nesting females typically construct and oviposit in combs with from 20 to 30 cells (West-Eberhard 1969). A *Polistes* foundress has at least two reproductive options besides solitary nest founding. She can join conspecific females in another nest or attempt to take over a nest initiated by a conspecific female (Reeve 1991). This behavior creates a series of advantages to the new nests, as productivity can increase and consequently, colony success can increase, offspring survival can improve in the case of dominant female death, as well as providing a more effective defense against natural enemies (West-Eberhard 1969; Itô 1985; Butignol 1992; Giannotti and Mansur 1993; Tannure and Nascimento 1999; Sinzato and Prezoto 2000; Tibbetts and Reeve 2003). During the colony's foundation (i.e. prior to eclosion of new adults), aggressive interactions happen among the nestmates, many times involving intense fights (West-Eberhard 1969; Gamboa and Dropkin 1979; Strasmmann 1989).

Colony productivity of the *Polistes* sp. (Latreille) in neotropical areas was already studied for different species, including *P. versicolor* (Gobbi and Zuchi 1985; Gobbi et al. 1993; Ramos and Diniz 1993), *Polistes lanio lanio* (Giannotti 1992), *Polistes cinerascens, Polistes canadensis* (Giannotti 1997; Santos and Gobbi 1998) and *Polistes simillimus* (Prezoto 2001). These studies corroborated the Michener (1964) paradox of an inverse relationship between the group size and per capita productivity.

The objective of this work was to characterize the foundation pattern, the productivity and the colony success of *P. versicolor* in anthropic environments.

## Material and Methods

The study was conducted from August 2003 to December 2004 in Juiz de Fora municipal district (21° 46′ S; 43° 21′ W, medium altitude of 678 m), Minas Gerais state, Southeastern Brazil, characterized by a high tropical climate according to the Koppen Scale. For obtaining data, the work was divided into three stages: foundation pattern characterization and nesting substrata, nest productivity analysis, and attendance and success of the colonies.

### Foundation pattern characterization and nesting substrata

For the information collection on the foundation pattern of *P. versicolor* colonies, weekly visits to the colonies took place at different places in the city of Juiz de Fora, preferably at the end of the afternoon (17 h), when the individuals were finishing their foraging activities, allowing a more precise counting of the number of foundresses present in the colony. During the visits, the following parameters were noted: number of females involved during the colony foundation phase (*n* = 100 nests) and substratum type used for the colony foundation (*n* = 192). In addition, behavioral information (*ad libitum* sensu Altmann 1974) exhibited by the individuals was obtained relating to the new nest construction process.

### Nest productivity analysis

For the productivity analysis, 37 *P. versicolor* nests collected at different places around the study area were sampled. The nests were dissected, and the information schematized in mappings in standardized leaves. For each nest, the following parameters were observed: total number of cells, total of productive cells, total of produced adults (by the counting of meconium layers deposited in the cells), number of adults produced per cell, and the ratio of produced adults/cells.

### Attendance and success of the colonies

The *P. versicolor* colonies were considered successful when they reached the post-emergence phase, according to the classification proposed by Jeanne (1972), with the production of at least one adult. A hundred colonies were followed from the foundation phase to the first adult's emergence and/or abandonment of the nest. The colony was considered unsuccessful when, for three consecutive visits, the complete absence of adults and immatures was observed, besides the lack of eggs postures and new cells construction.

### Statistical analyses

In order to verify the difference existence among the categories of nesting substrata used by *P. versicolor*, the χ^2^ test was applied. The Spearman correlation test was used to correlate the total number of cells and the total number of adults produced in the sampled nests. The tests were completed using Bioestat 4.0.

## Results and Discussion

### Foundation pattern characterization of the colonies

**Figure 1.**
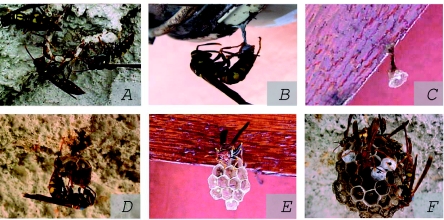
*Polistes versicolor* colony contruction pattern in an anthropic environment, Juiz de Fora, Minas Gerais state, Southeastern Brazil. A = place inspection for foundation, B = oviposition behavior in the first cell, C = first cell with circular format and egg, D = construction behavior by the female in the solitary foundation, E = hexagonal format of cells in the solitary foundation, and F = foundress association. High quality figures are available online.

The *P. versicolor* nests were built with chewed vegetable material, which was added in the peduncle and in the cells, resulting in a grayish coloration. Before beginning the construction, the foundress made recognition flights, and inspected the structures to be used for the nest (Figure 1A). This construction pattern is similar to those described for other *Polistes* species (West-Eberhard 1969; Reeve 1991; Karsai and Theraulaz 1995).

Once the place for the new colony foundation was established, the nest construction began, starting with the peduncle. This was followed by construction of the first cells with a circular format (Figures 1B and 1C), and as the number of cells increased, these assumed hexagonal outlines (Figures 1D and 1E). With the increase in number of cells, the peduncle is reinforced through the addition of chewed vegetable fiber. Colony contact to the substratum was reduced by the fine peduncle that represents an defensive adaptation against predatory pressure from ants (Jeanne 1975).

Six behavioral actions exhibited by *P. versicolor* during nest construction were identified, corroborating the description of the genus by Evans and West-Ebehard (1970):

1) Inspection of sites for nest foundation which is characterized by flights close to the selected area. The foundress touches the substratum with the antennae.

2) Construction of the pedicle: vegetable fiber is chewed with saliva, which is then attached to the substrate for construction of the peduncle in thread form.

3) Initial cell construction: after construction of the peduncle, the initial cell is constructed with a circular format. During this activity, the female constantly touches the cell sides with the antennae.

4) Construction of peripheral cells: new cells are added around the initial cell, assuming hexagonal outlines as they are attached to neighboring cells.

5) Cell prolongation: as larvae develop, chewed vegetable fiber is added to the extremities of the cells, elevating their height.

6) Peduncle invigoration: as cell number increases, the peduncle is reinforced with construction material, which makes it thicker to support the nest as it enlarges.

### Substrata used for nesting

Six substrata categories used for nesting by *P. versicolor* were recognized: gypsum plaster (*n* = 115; 59.9%), metal (*n* = 35; 18.3%), wood (*n* = 30; 15.6%), several materials (*n* = 8; 4.2%), glass (*n* = 2; 1.0%) and vegetation (*n* = 2; 1.0%) (Table 1). Occurrence of nests on gypsum plaster was significantly larger (p < 0.0001; chi-square test) relative to the other substrates. These nests were found mainly at high places in buildings, where the nests presumably obtained greater protection from human interference, weather, and direct solar irradiation.

A large number of *Polistes* species use human constructions as nesting substratum (Fowler 1983; Butignol 1992; Giannotti and Mansur 1993); although they can also use natural environment, such as plants and termite colonies (Makino 1985; Henriques et al. 1992; Claperton 2000; Sinzato and Prezoto 2000). The results of this study demonstrated the *P. versicolor* synantropism in relation to the constructions with little human interference, which is a behavior already described for the species (Butignol 1992; Sinzato and Prezoto 2000), as well as for *P. lanio* (Giannotti 1992) and *P. simillimus* (Prezoto 2001).

The vegetation present in the anthropic environment was used by a small number of colonies, that might be attributed to the fragility of the plants, that consisted of species used for gardening that did not offer appropriate support for the nest, and exposed the nest to the stress of weather.

Lima et al. (2000) studied the substrata used by the social wasps in an area close to this study area, and they verified that the *Polistes* species found nested preferably in human constructions; finding nests in the vegetation was rare. Butignol (1992) observed that the plants used as substratum by *P. versicolor*, in Florianópolis, south of Brazil, had perennial leaves, such as *Acacia podzarilifolia, Fucreasea gigantea* and *Acalipa wilkesianae*. The use of plants as nesting substratum in anthropic environments was also observed by Giannotti (1995), who recorded nine *Polistes subsericeus* colonies in a single *Pandanus veitichi* (Pandanaceae) plant. The author suggested that this plant offers a protected and criptic shelter for this species' colonies. Although the anthropic environment offers nesting resources, some species demonstrate preference for nesting in the natural environment, as observed by Claperton (2000) for *Polistes humilis* and *Polistes chinensis antennalis*.

**Table 1.**
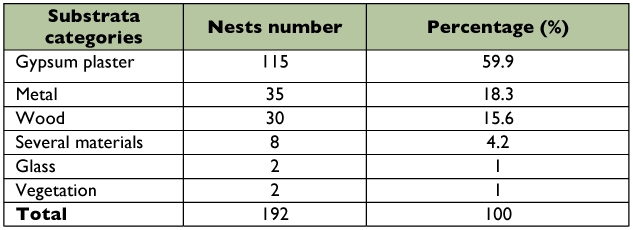
Substrata categories used for nesting by *Polistes versicolor* in an antropic environment in southeast Brazil.

*P. versicolor* foundations were also registered at places used previously by other conspecific colonies (*n* = 25). This behavior was also registered by Giannotti (1992) for 12 *P. lanio* colonies and by Prezoto (2001) for 13 *P. simillimus* colonies. Prezoto (2003) suggested that this behavior reflects the ability to perceive and analyze information regarding the appropriate nesting sites perhaps including odor left by an old colony that could be an incentive for nest foundation.

### Colony productivity

It was verified that the medium number of cells produced by *P. versicolor* nests was 244.2 ± 89.5 (100–493); the unproductive cells percentage was 44.5% (13.6–72.2%), and the average number of adults produced per nest was 171.67 ± 109.94 (37–660) (Table 2). The ratio of individuals produced per cell was 0.66, registering a maximum of six uses in a single cell.

Gobbi and Zucchi (1985) studied the productivity of *P. versicolor* in an anthropic area, the municipality of Ribeirão Preto, São Paulo state, Southeastern Brazil, in 1975 and 1976, and they observed a variation in the number of cells produced (191.80 ± 56.51 and 221.67 ± 132.05, respectively), irrespective of the number of adults produced those years (98.70 ± 40.09 in 1975 and 174.61 ± 153.20 in 1976). Based on these results, the authors suggested that *P. versicolor* may present short cycle colonies (some months), as information was registered in 1975 and long cycle colonies (around a period of a year), as registered for those colonies studied in 1976, what favored a larger productivity of the latter. The results found in the present study are similar to those verified by Gobbi and Zucchi (1985) for the colonies studied in 1976. This suggests that the colonies studied presented a long cycle, which is a common occurrence in anthropic areas.

**Table 2.**
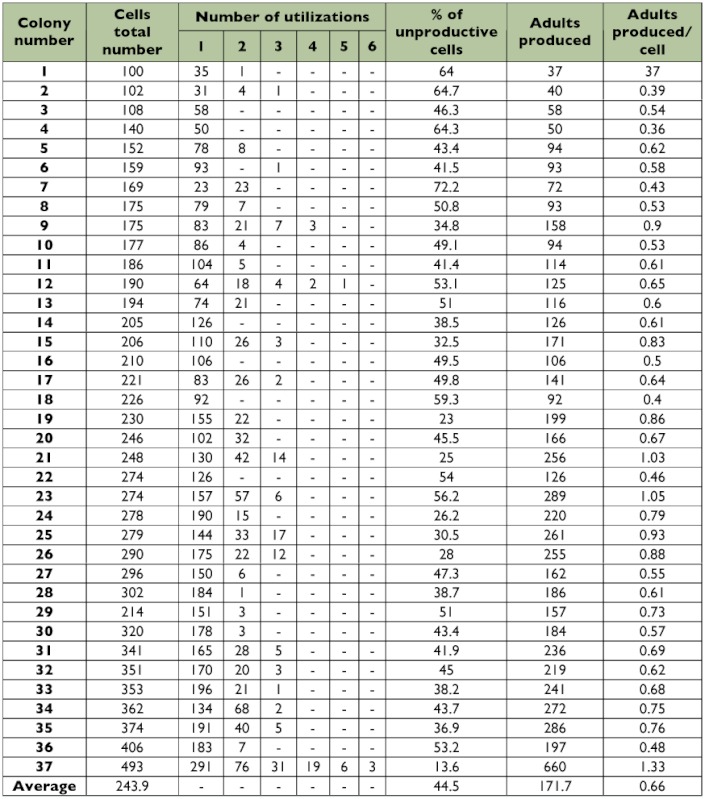
Comparative data of 37 nests' productivity by *Polistes versicolor* colected in an antropic environmet in southeast Brazil.

In a comparative work, Gobbi et al. (1993) studied *P. simillimus* and *P. versicolor* productivity, and they verified an average of 391.3 ± 302.34 and 80.0 ± 114.88 cells per nest, respectively. About 60% of the *P. simillimus* colonies used the cells to produce two adult generations, while for *P. versicolor*, only 25% of the colonies used cells more than once. However, for *P. versicolor* the authors found cells that produced three generations. Giannotti (1997) verified that *P. cinerascens* nests in Rio Claro, São Paulo, included 102.9 cells and 94.2 individuals on average per nest, whose adult/cell ratio was 0.8 and some cells produced up to four individuals. Santos and Gobbi (1998), in a Savanna area in Bahia, verified that the *P. canadensis* nests possess 184.17 (29–477) cells on average, which produce 163 (10–576) individuals on average, with a single cell able to produce up to four individuals.

According to Prezoto (2001), *P. simillimus* produces nests with about 337.28 (8–1325) cells, and 57.21% (4.38–95.46%) of these are unproductive, reflecting a small number of reutilizations (36% for two uses and 24% for three). He also affirms that *P. simillimus* nests can produce 256.36 (1–1355) adults on average, with a ratio of 0.44 (0.04–1.02) adults produced per cell, a smaller value than the one found for *P. versicolor* in our study.

There was a positive correlation (r = 0.8498; p < 0.001, Spearman correlation test) between the total number of cells and the total number of adults produced by the *P. versicolor* nests. As the colonies grew, there was an increase in the number of adults produced, as well as more cell reutilization, while for other species the number of reutilizations is smaller, as in *P. simillimus* (Prezoto 2001). Ramos and Diniz (1993), also studying *P. versicolor* in an urban area of Brasilia, observed a positive correlation (r = 0.902, p < 0.001) between the number of cells and the number of adults produced, and the cells were used up to four times.

The *P. versicolor* unproductive cells were concentrated on periphery of the comb, which was also noted for *P. canadensis* (Santos and Gobbi 1998) and *P. simillimus* (Prezoto 2001), and the cells with the largest number of utilizations were located in close proximity to the peduncle and in the central nest area, that are the oldest part of the comb. This disposition can work as a strategy against the predatory pressure, parasitism and reproductive conflicts, all mentioned by Gobbi et al. (1993) as factors that impose limits on the number of cells built in *Polistes* nests.

### Colony success

Most of the new *P. versicolor* nests were founded by a foundresses association (*n* = 68) (Figure 1F), which was responsible for the largest number of successful colonies (*n* = 35; 51.5%). However, foundation by solitary females presented smaller incidence (*n* = 32), whose success was even smaller (n= 3; 9.4%).

Studies accomplished at other places in Brazil describe foundress association as a foundation type commonly observed for *P. versicolor* (Itô 1985; Butignol 1992; Giannotti and Mansur 1993; Ramos and Diniz 1993; Tannure and Nascimento 1999; Sinzato and Prezoto 2000). Females association is also a common strategy in other neotropical species such as *Polistes ferreri* (Tannure and Nascimento 1999), *P. canadensis* (Itô 1985) and *P. lanio* (Giannotti 1992). However, Prezoto (2001) observed that the foundation by a single female constitutes 56.3% of *P. simillimus* foundations, with success of 37.09% of them. In spite of that, the author observed that, even being the smallest part of the total foundations, the foundress association was responsible for the largest number of successful colonies in *P. simillimus*.

Itô (1985) observed that in colonies with a larger number of foundresses the duration of the pre-emergence phase period was reduced and the group size was related positively with the number of built cells, and these colonies were more productive. However, the author found that productivity of individual foundresses was lower in these colonies. Therefore, the females' association during the foundation is interpreted as an optimization strategy, in which ecological pressures such as parasitism and usurpation, social pressures such as the effects of the ergonomic synergism, and the increase of survival levels are all associated (West-Eberhard 1969; Gamboa 1978; Gibo 1978; Strassmann 1981, 1989; Itô 1985; Reeve 1991; Wenzel 1996).

The high failure number (90.06%) of the colonies founded by a single female *P. versicolor* in the present study occurred mainly because the foundress abandoned the nest during the initial colony establishment phase, before the larvae appeared. This same phenomenon was described by Tannure and Nascimento (1999) for the same species. It is believed that this behavior is associated with the fact that the wasps migrate in search of association with other foundresses. Other factors as foundress death or disappearance and dominance disputes also promote colony failure in *Polistes* species (Reeve 1991; Giannotti and Mansur 1993; Tannure and Nascimento 1999; Prezoto 2001).

This study's results demonstrate that *P. versicolor* nesting behavior is very similar to that described for other *Polistes* species. In an anthropic environment, *P. versicolor* exhibited a preference for artificial substrata for nesting, which probably provides larger longevity for the nests due to protection from the stress of weather. In this type of environment, usually a group of females found their nests in different climatic situations, which results in production of colonies of various sizes and also causes a different productivity among them. Although these results enlarge knowledge on the *P. versicolor* foundation pattern in anthropic environments, there are many subjects needing further study mainly to increase knowledge about nesting behavior of other neotropical *Polistes* species.
